# MED5/355: Using Web-technology for Asynchronous Telemedicine Consulting

**DOI:** 10.2196/jmir.1.suppl1.e48

**Published:** 1999-09-19

**Authors:** Y Reviakin, A Sukhanov

**Affiliations:** ^1^Space Biomedical Centre for Training and ResearchMoscowRussia

**Keywords:** Remote Consultation, Databases, Web Technology

## Abstract

**Introduction:**

Common telemedicine consultations can be divided in two classes: real-time telemedicine consultations and asynchronous telemedicine consultations. The advantage of real-time consultations is obvious - this is a natural discussion between physicians, which may be realised on the basis of desktop videoconferences. But the problems are also obvious: the necessity of additional hardware and the elevated demands for channel bandwidth. Because of the latter, the use of asynchronous telemedicine consultations is more preferable in most cases.

**Methods:**

We can consider asynchronous telemedicine consultations as a special form of a discussion forum, very familiar tool for users' collaboration in global networks. Currently, there exist many software products that facilitate the discussion forums using Web-technology and Internet standards. After a period of research and analysis, we have taken the decision to use as our development basis the Lotus Notes/Domino server. The following characteristics of Lotus Notes can explain our decision:

- Ready-to-run discussion forum model
- Mechanisms for access control,
- Build-in integration with Web-technology,
- Visual tools for database design.

**Results:**

The first version of Web-server for telemedicine consultation "Telemedicine concilium" using Lotus Notes/Domino technology is already in use. The main form, describing clinical case description documents, has the two main parts: description of patient vital status/systems and section of multimedia upload files (attached files with clinical investigation data). The clinical cases database is currently containing about 30 clinical cases. Approximately 40 physicians not only from Moscow, but also from other regions of Russia and from other countries of ex-USSR were registered as users of server.

**Discussion:**

The current operation of the Lotus Notes server has proven the efficiency and attraction of the proposed technology. However, some problems have been detected: the Web-interaction mode is not available for all users, while some of them prefer to use e-mail for information exchange. Also, the form for the clinical case descriptions must be differentiated for different clinical cases or diverse category of patients (adults, children).

